# Echocardiographic Hemodynamics After Aortic Valve Replacement, Wheat, and Bentall Procedure

**DOI:** 10.3390/jcm14217627

**Published:** 2025-10-27

**Authors:** Wakana Niwa, Yoshiyuki Takami, Atsuo Maekawa, Koji Yamana, Kiyotoshi Akita, Kentaro Amano, Kazuki Matsuhashi, Yasushi Takagi, Tomonobu Abe

**Affiliations:** Department of Cardiac Surgery, Fujita Health University School of Medicine, Toyoake 470-1192, Japan

**Keywords:** aortic prosthetic valve, aortic valve replacement, echocardiography, Wheat procedure, Bentall procedure

## Abstract

**Background**: Compared with isolated aortic valve replacement (AVR), echocardiographic hemodynamics after Wheat and Bentall procedures, both involving replacement of the proximal ascending aorta with a smaller-diameter graft, have been less thoroughly investigated. **Methods:** We analyzed 213 patients who received 21 mm or 23 mm aortic bioprostheses (AVR, *n* = 138; Wheat, *n* = 43; Bentall, *n* = 32). Transthoracic echocardiography was performed before and after surgery, and the proximal ascending aortic area (Aa) was assessed using contrast-enhanced computed tomography. **Results:** The maximal pressure gradient (PG max), derived from the simplified Bernoulli equation, was significantly lower in the Bentall group, whereas pressure recovery (PR), calculated using Voelker’s equation, was lower in the AVR group. A smaller Aa was associated with a higher PG max in the AVR group. The Bentall group exhibited significantly lower energy loss (EL). In propensity score-matched analyses to minimize potential confounding factors, the AVR group showed a significantly lower PR and higher EL than the Wheat group; a significantly higher PG max, lower PR, and higher EL than the Bentall group; and a significantly similar PR but lower EL in the Bentall group compared with the Wheat group. **Conclusions:** Although limited to bioprosthetic valves, caution is warranted when interpreting echocardiographic PG max after AVR in patients with a small ascending aorta. However, overestimation of PG max was not observed in either the Wheat or Bentall groups, even though both demonstrated higher PR and lower EL compared with the AVR group.

## 1. Introduction

The hemodynamic performance of aortic valve (AV) prostheses is typically evaluated by echocardiography, using the simplified Bernoulli equation to calculate transprosthetic maximal and mean pressure gradients (PGs) [1]. The simplified Bernoulli equation assumes that the increase in convective velocity across the AV is entirely due to pressure loss from AV stenosis, while neglecting local acceleration, viscous forces, and proximal left ventricular outflow tract velocity. In addition to this simplification, failure to account for pressure recovery (PR) is a well-described cause of the discrepancy between echocardiographic and catheterization-derived pressure PGs in AV prostheses [2,3].

PR is a phenomenon reflecting the dynamic interplay between kinetic and potential energy across the AV [1]. As blood flows across the AV, potential energy is converted into kinetic as flow velocity accelerates. Distal to the stenosis, some of this kinetic energy is reconverted into potential energy, resulting in a partial restoration of pressure detectable by catheterization—this is referred to as PR. Greater PR leads to an overestimation of PG by echocardiography compared to those measured by catheterization. PR is more pronounced in conditions such as high-flow states, small non-compliant aortas, and geometrical configurations that prevent the normal outward turbulent expansion of blood into the sinuses of Valsalva [4,5,6].

The flow characteristics of AV prostheses may differ following simple AV replacement (AVR), AVR with ascending aortic replacement (Wheat procedure), and the Bentall procedure, potentially affecting PR. In patients undergoing the Wheat and Bentall procedures, proximal aortic prosthetic grafts with smaller diameters and increased impedance may contribute to greater PR and an overestimation of the echocardiographic pressure gradient (PG) across the AV prosthesis. In the present study, we retrospectively compared the echocardiographic evaluation of AV prostheses following AVR, Wheat, and Bentall procedures.

## 2. Materials and Methods

### 2.1. Ethical Statement

This retrospective study to analyze single-center data was approved by the Fujita Health University Ethics Committee for clinical study and was conducted according to the ethical guidelines published by the Ministry of Health and the Helsinki Declaration. The approval number is HM24-607, 22 December 2024, including a waiver of informed consent.

### 2.2. Study Patients

**Figure 1:**
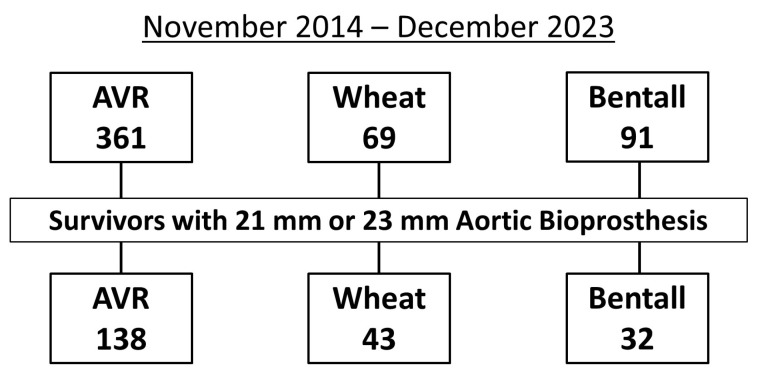
Flowchart showing the inclusion criteria for study patients with 21 mm or 23 mm aortic bioprostheses after aortic valve replacement (AVR), Wheat procedure (AVR with ascending aortic replacement), and Bentall procedure.

Between November 2014 and December 2023, 361 patients underwent AVR, 69 underwent the Wheat procedure, and 91 underwent the Bentall procedure. Of these, the study population (*n* = 213) consisted of 138 patients who underwent AVR, 43 who underwent the Wheat procedure, and 32 who underwent the Bentall procedure, all of whom received 21 mm or 23 mm aortic bioprostheses and were discharged alive after surgery (Figure 1).

The Bentall group underwent the aortic root replacement using a valve conduit according to the Svensson modification [7]. The distant left main coronary ostium was reattached to the ascending conduit via a 10 mm interposition prosthetic graft. The right coronary artery button was mobilized and conventionally reattached.

### 2.3. Echocardiography

Echocardiography was performed in the study patients at the outpatient clinic between 6 months and 1 year after surgery to remove the effects of the surgery. The following left ventricular (LV) systolic and diastolic parameters were assessed: LV end-diastolic dimension (LVDd), LV end-systolic dimension (LVDs), LV ejection fraction (LVEF), LV mass (LVM), and relative wall thickness (RWT). The LVEF was measured from the apical 4- and 2-chamber images using the biplane method of disks. LVM was calculated according to the following formula: LVM (g) = 0.8 {1.04 [([LVDd + interventricular septum thickness + LV posterior wall thickness]^3^ − LVEDd^3^)]} + 0.6. The LVM index was calculated as LVM/body surface area (BSA). RWT was calculated with the following formula: RWT = (2 × posterior wall thickness)/LVDd.

Maximal transvalvular pressure gradients (PG max) were calculated with the use of the simplified Bernoulli equation from the aortic velocity obtained by multiwindow continuous-wave Doppler interrogation. The effective orifice area (EOA) of the aortic bioprosthesis was calculated using the continuity equation as the stroke volume measured in the LV outflow tract (LVOT) divided by the aortic velocity–time integral. LVOT stroke volume was calculated as the product of the LVOT cross-sectional area and LVOT velocity–time integral measured by pulsed-wave Doppler. The pulsed-wave Doppler sample volume was located just apical to the prosthetic valve stent or sewing ring. The LVOT diameter was measured from the outer-to-outer border of the stent or sewing ring [8].

The following equations, using Doppler-derived parameters, whose problem was solved by developing the Voelker equation [9], were used to calculate PR, the PR index (PRI), energy loss (EL), and the energy loss coefficient (ELCo) referring to the corresponding “functional valve orifice area” [10,11]: PR (mmHg) = 4Vmax^2^ × 2EOA/Aa × (1 − EOA/Aa); PRI = 2(EOA/Aa − (EOA/Aa)^2^); EL (mmHg) = 4Vmax^2^ × (1−EOA/Aa)^2^; and ELCo (cm^2^/m^2^) = (EOA × Aa)/(Aa − EOA)/BSA, where Aa is the cross-sectional area of the aorta.

To analyze the Doppler data, the average of at least three cardiac cycles was used (10 cycles for patients with atrial fibrillation). Patients with heart rates below 40 or above 120 beats/min were excluded.

The proximal ascending aortic diameter (Ad) and area (Aa) were measured about 5 mm above the sinotubular junction on preoperative contrast CT scans in the patients undergoing AVR. This localization was previously recommended [3,4], because at this position, the blood flow should be laminar again, and thus the pressure recovery should be completed. In those undergoing Wheat and Bentall procedures, Ad and Aa, which were diameter and area of the prosthetic graft, were obtained on postoperative contrast-enhanced CT scans.

### 2.4. Statistical Analysis

All statistical analyses were executed using software (EZR, available online [12]). A *p* value less than 0.05 was deemed significant for all tests. Categorical variables were expressed as count and percentage and compared with the chi-square test. Continuous variables were expressed as means ± standard deviations and compared using analysis of variance (ANOVA) or the Kruskal–Wallis rank sum test with appropriate post hoc tests (Tukey HSD or Steel–Dwass, respectively), according to the Kolmogorov–Smirnov test to check for normal distribution.

To reduce potentially confounding factors, propensity score matching was performed to balance risk factors between the groups using 1:1 nearest-neighbor matching with caliper 0.1. To assess covariate balance (potential confounders), the standardized mean difference was used, and logistic regression was employed for propensity score matching.

## 3. Results

### 3.1. Baseline Characteristics

As shown in Table 1, the patients of the Bentall group were significantly younger than those of the AVR and Wheat groups. The AVR group had significantly higher prevalences of diabetes mellitus and atrial fibrillation on electrocardiography than the Wheat and Bentall groups.

### 3.2. Operative Data

As shown in Table 2, there was a significantly lower prevalence of AV stenosis as a primary disease of the AV in the Bentall group. In the AVR group, 36% of the patients underwent isolated AVR, while 64% underwent AVR with concomitant procedures, including mitral valve replacement/repair and coronary artery bypass grafting. The Bentall group included more patients undergoing surgery under circulatory arrest and therefore showed a significantly longer time for operation, cardiopulmonary bypass, and cardiac arrest. Also, the lowest body temperature during surgery was significantly lower in the Bentall group. As shown in Table 2, the kinds and sizes of the aortic bioprostheses and vascular grafts were not significantly different in the three groups. Triplex grafts (Terumo Corp, Tokyo, Japan), with sizes of 24, 26, and 28 mm, were mostly used in the Wheat and Bentall groups.

### 3.3. Echocardiographic Data

**Figure 2:**
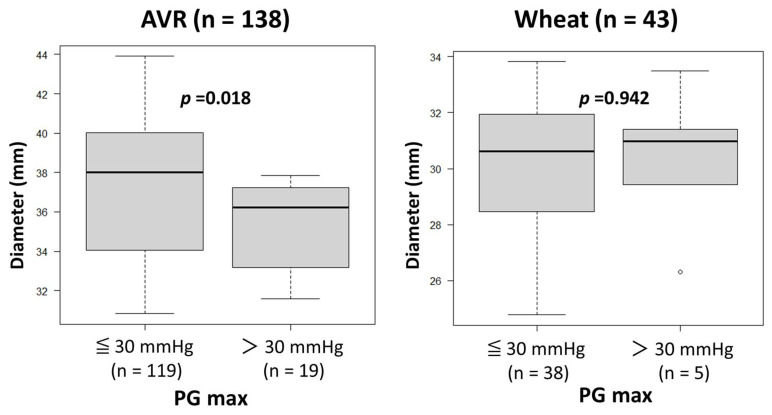
Comparison of the ascending aortic diameters between the patients with maximal transvalvular pressure gradients (PG max) of ≤30 mmHg and those with a PG max of >30 mmHg after surgery in aortic valve replacement (AVR) and Wheat groups.

The heart rates during Doppler examination were 73 ± 16 beats/min (range, 45–112). No patients, including those with atrial fibrillation, were excluded from the study due to unsatisfactory Doppler data. As shown in Table 3, preoperative echocardiography showed similar variables other than LVEF, which was significantly lower in the Bentall group than in the AVR and Wheat groups. Postoperative echocardiography showed significantly lower PG max values in the Bentall group than in the AVR and Wheat groups, while EOAs were similar among the three groups. There were no patients with PG max > 30 mmHg in the Bentall group. A comparison of the patients with PG max > 30 mmHg and ≤30 mmHg revealed that the ascending aortic diameter was significantly smaller in those with PG max > 30 mmHg than in those with PG max ≤ 30 mmHg in the AVR group (Figure 2). However, in the Wheat group, there were no significant differences in aortic diameter between those with PG max > 30 mmHg and those with PG max ≤ 30 mmHg.

Since the aorta diameter and Aa measured on CT angiograms were significantly larger in the AVR group than in the Wheat and Bentall groups, PR and PRI were significantly lower in the AVR group than in the other two groups. The Bentall group demonstrated a significantly lower EL and higher ELCo than the other two groups (Table 3).

### 3.4. Propensity Score-Matched Comparisons

To compare the AVR and Wheat groups, propensity score matching was performed, controlling diabetes and atrial fibrillation to generate a balanced cohort made up of 38 pairs (Table 4). Both matched groups were similar across all demographic characteristics and preoperative echocardiographic data. Since the Aa was significantly larger in the AVR group, the AVR group showed significantly lower PR and higher EL values than the Wheat group.

To compare the AVR and Bentall groups, propensity score matching was performed, controlling for diabetes, atrial fibrillation, and preoperative LVEF to generate a balanced cohort made up of 24 pairs (Table 5). Postoperative echocardiography showed a significantly higher PG max in the AVR group than in the Bentall group. Since the Aa was significantly larger in the AVR group, the AVR group showed significantly lower PR, lower PRI, and higher EL values than the Bentall group.

To compare the Wheat and Bentall groups, propensity score matching was performed, controlling for only preoperative LVEF to generate a balanced cohort made up of 21 pairs (Table 6). Since the Aa was similar in both groups, PR and PRI were also similar, but EL was significantly lower, and ELCo was significantly higher in the Bentall group than in the Wheat group.

## 4. Discussion

**Figure 3:**
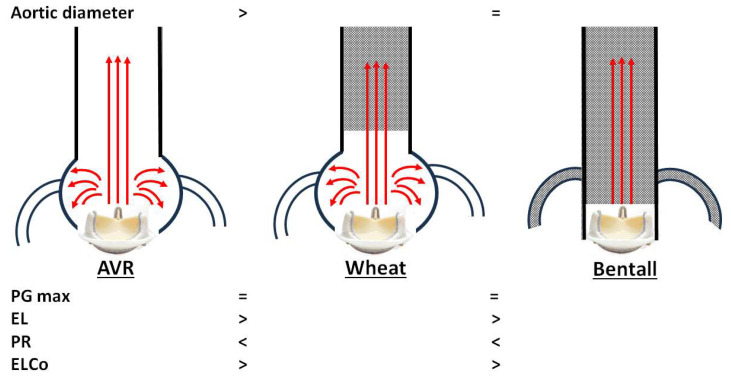
Conceptual summary of our echocardiographic comparison of aortic valve prostheses after aortic valve replacement (AVR), Wheat, and Bentall procedures. PG max, maximal transvalvular pressure gradient; EL, energy loss; PR, pressure recovery; ELCo, energy loss coefficient.

Our main echocardiographic findings comparing AV prostheses after AVR, Wheat, and Bentall procedures are summarized as follows, although these are limited to bioprosthetic valves (Figure 3):

1. Unexpectedly, overestimation of PG max was not observed in either the Wheat or Bentall groups, despite replacement of the ascending aorta with a smaller vascular graft.
2. In the AVR group, a smaller Ad was associated with a higher PG max after AVR. Patients with PG max > 30 mmHg had a significantly smaller Ad than those with PG max ≤ 30 mmHg.
3. Both the Wheat and Bentall groups demonstrated higher PR and lower EL values compared with the AVR group.

PR downstream of the AV is an important factor affecting the calculation of PG across the valve and thereby the estimation of the AV area [13]. PR represents the conversion of kinetic energy back into potential energy, which may lead to overestimation of echocardiographic PG. This phenomenon has been demonstrated experimentally [14], observed clinically [15], and precisely calculated using Doppler ultrasound based on Voelker’s equation [9]. The Aa and the presence of the sinus of Valsalva are critical determinants of PR. While the hemodynamic effects of surgical Aa reduction by graft replacement in Wheat and Bentall procedures have received little attention, valve performance and PR have been extensively studied in transcatheter aortic valve replacement (TAVR) [16,17,18]. The Edwards SAPIEN 3 (balloon-expandable valve) generates laminar flow, resulting in greater convective acceleration, increased PR, and higher echocardiographic PG. In contrast, the Medtronic Evolut R (self-expandable valve) produces slightly turbulent flow due to its stent structures that interact with the ascending aorta, resulting in less convective acceleration, lower PR values, and reduced echocardiographic PG.

One possible explanation for the absence of PG max overestimation in the Wheat and Bentall groups may be the unexpectedly high compliance of the Triplex graft used for ascending aortic replacement. The Triplex graft consists of three layers: an inner uncoated woven Dacron graft, an outer expanded polytetrafluoroethylene layer, and a middle non-biodegradable styrene elastomer resin layer [19]. Recent reports have described elongation of Triplex grafts, speculated to be due to reduced adhesion formation with surrounding tissues and insufficient stability and fixation of the prosthesis [20,21]. In addition, thermal crimping treatment is applied to induce a wavy textile configuration in the Dacron layer. This crimping helps preserve the tubular shape while providing bending flexibility and axial compliance [22].

As demonstrated in Figure 2, a smaller Aa increases PG max in the AVR group. Therefore, careful interpretation of echocardiographic PG max is warranted in AVR patients, especially with a smaller ascending aorta.

Our third finding is that the Bentall procedure may be associated with reduced LV work, as suggested by lower EL and higher ELCo values compared with the AVR and Wheat procedures. ELCo has been established as a useful parameter for stratifying patients with AV stenosis into high-, moderate-, or low-risk categories (with ELI < 0.6 cm^2^/m^2^ indicating severe aortic stenosis) [10]. A more accurate hemodynamic model considers not only velocity and pressure but also heat, as turbulent flow and frictional resistance across a valve result in energy loss as heat. Patients with a higher ELCo, reflecting less turbulence and shear stress, have been reported to show improved survival after TAVR [23]. The hemodynamic findings in the Bentall group, which lacks the sinus of Valsalva, indicate a valve–vessel interaction with more laminar flow. This led to significant PR gain and improved ELCo, essentially reflecting an increased functional AV orifice area.

The present study has several limitations. First, this was a single-center, retrospective observational study with a relatively small sample size, which may limit the generalizability of our findings. Second, although the Aa immediately distal to the sinotubular junction is critical for PR measurements, we did not account for the diameter or cross-sectional area of the sinus of Valsalva in the AVR and Wheat groups, which may also affect flow dynamics. Third, valve types were not uniform across the groups, and differences in valve material may have influenced the results. Fourth, the underlying aortic valve pathology differed among the groups: aortic stenosis was predominant in the AVR and Wheat groups, while aortic regurgitation was predominant in the Bentall group. Fifth, we did not take into consideration the viscosity of blood, which is also important for fluid dynamics through the AV prosthesis. Sixth, the AVR group included more patients with atrial fibrillation. Although propensity score matching was performed to minimize this potential confounding effect, it may still have influenced the Doppler measurements. Finally, the clinical implications of the present findings remain uncertain, and further investigation is required.

## 5. Conclusions

Although the present findings are limited to bioprosthetic valves, a smaller diameter of the ascending aorta was closely related to an increased PG max on echocardiography in the AVR group. In comparison to the AVR group, both the Wheat and Bentall groups showed a higher PR and lower EL. However, overestimation of PG max on echocardiography was not observed in both the Wheat and Bentall groups.

## Figures and Tables

**Table 1:** Patient characteristics of the complete cohort.

Variables	AVR (*n* = 138)	Wheat (*n* = 43)	Bentall (*n* = 32)	*p* Value
Age, years	74 ± 6	74 ± 8	71 ± 7	<0.001
Male gender, *n* (%)	101 (73)	31 (72)	24 (75)	0.87
Height, cm	161 ± 8	163 ± 9	163 ±8	0.89
Weight, kg	60 ± 11	60 ± 11	61 ± 12	0.84
Hypertension, *n* (%)	109 (78)	32 (74)	25 (78)	0.65
Hyperlipidemia, *n* (%)	75 (54)	21 (49)	15 (47)	0.23
Diabetes mellitus, *n* (%)	32 (23)	7 (16)	6 (19)	0.02
Previous stroke, *n* (%)	14 (10)	2 (5)	3 (9)	0.68
Lung disease, *n* (%)	32 (23)	8 (19)	6 (19)	0.67
Smoking habit, *n* (%)	68 (49)	20 (47)	15 (47)	0.79
Liver disease, *n* (%)	5 (4)	1 (2)	0	0.15
Chronic kidney disease, *n* (%)	62 (45)	16 (37)	13 (41)	0.32
Peripheral artery disease, *n* (%)	10 (7)	1 (2)	0	0.25
Atrial fibrillation, *n* (%)	28 (20)	4 (9)	1 (3)	<0.001
CTR on X-ray, %	54 ± 7	53 ± 8	55 ± 7	0.71

Data are presented as means ± standard deviations or frequencies (percentages). AVR, aortic valve replacement; CTR, cardiothoracic ratio.

**Table 2:** Operative data in the complete cohort.

Variables	AVR (*n* = 138)	Wheat (*n* = 43)	Bentall (*n* = 32)	*p* Value
Urgency or emergency	4 (3)	1 (2)	2 (6)	0.82
Redo surgery	2 (1)	2 (5)	2 (6)	0.66
Primary disease of aortic valve				
Aortic valve stenosis	81 (57)	27 (62)	4 (13)	<0.001
Aortic valve regurgitation	57 (43)	16 (38)	28 (87)	
Bicuspid aortic valve	36 (26)	14 (33)	9 (28)	0.71
Isolated AVR	50 (36)	0	0	-
Concomitant procedures	88 (64)			
MVR	12 (9)	1 (2)	2 (6)	0.24
MVP	17 (12)	1 (2)	3 (9)	0.18
CABG	41 (28)	10 (23)	11 (34)	0.29
Operation, min	363 ± 85	438 ± 131	528 ± 104	<0.001
Cardiopulmonary bypass, min	185 ± 50	222 ± 55	297 ± 66	<0.001
Cardiac ischemia, min	127 ± 36	160 ± 35	222 ± 46	<0.001
Lowest body temperature, °C	31 ±2	25±3	25±4	<0.001
Circulatory arrest, *n* (%)	4 (3)	34 (79)	16 (50)	<0.001
AVR bioprosthesis				0.37
Avalus		27 (63)	22 (69)	
Magna ease	36 (26)	5 (12)	5 (16)	
Inspiris	26 (19)	6 (14)	2 (6)	
Mosaic	2 (1.5)	3 (7)	3 (9)	
Epic	2 (1.5)	2 (5)		
AVR size				0.54
21 mm	79 (57)	23 (53)	12 (38)	
23 mm	59 (43)	20 (47)	20 (62)	
Vascular prosthesis				0.94
Triplex		42 (98)	32 (100)	
J Graft		1 (2)		
Vascular prosthesis size				0.24
22 mm		1 (2)		
24 mm		8 (19)	7 (22)	
26 mm		12 (28)	18 (56)	
28 mm		17 (40)	7 (22)	
30 mm		4 (9)		
32 mm		1 (2)		

Data are presented as means ± standard deviations or frequencies (percentages). AVR, aortic valve replacement; MVR, mitral valve replacement; MVP, mitral valve plasty; CABG, coronary artery bypass grafting.

**Table 3:** Echocardiographic data of the complete cohort.

Variables	AVR (*n* = 138)	Wheat (*n* = 43)	Bentall (*n* = 32)	*p* Value
Preoperative				
LVDd, mm	45 ± 7	47 ± 7	48 ±10	0.32
LVDs, mm	32 ± 9	31 ± 8	36 ± 12	0.12
LVEF, %	55 ± 10	56 ± 9	50 ± 11	0.02
LVMI, g/m^2^	109 ± 33	102 ± 27	121 ± 56	0.42
RWT	0.49 ± 0.10	0.50 ± 0.07	0.50 ± 0.08	0.72
Postoperative				
Aorta area, mm^2^	1074 ± 236	746 ± 184	696 ± 106	<0.01
Aorta diameter, mm	36.7 ± 4.4	30.6 ± 3.4	29.7 ± 2.3	<0.01
PG max, mmHg	23.3 ± 7.1	21.6 ± 7.7	17.8 ± 83	<0.01
EOA, cm^2^	1.38 ± 0.25	1.38 ± 0.26	1.46 ± 0.30	0.643
PR, mmHg	5.20 ± 1.75	5.75 ± 2.96	6.47 ± 2.19	0.034
PRI	0.23 ± 0.06	0.31 ± 0.06	0.34 ± 0.06	0.034
EL, mmHg	17.5 ± 5.9	14.5 ± 6.4	10.7 ± 5.1	0.026
ELCo, cm^2^/m^2^	1.00 ± 0.22	1.06 ± 0.25	1.20 ± 0.33	0.029

Data are presented as means ± standard deviations. LVDd, left ventricular end-diastolic dimension; LVDs, left ventricular end-systolic dimension; LVEF, left ventricular ejection fraction; LVM, left ventricular mass; RWT, relative wall thickness; PG max, maximal transvalvular pressure gradient; EOA, effective orifice area; PR, pressure recovery; PRI, pressure recovery index; EL, energy loss; ELCo, energy loss coefficient.

**Table 4:** Propensity score-matched comparisons between AVR and Wheat groups.

Variables	AVR (*n* = 38)	Wheat (*n* = 38)	*p* Value
Patient characteristics			
Age, years	72 ± 8	74 ± 8	0.273
Gender, male, *n* (%)	25 (66)	25 (66)	1.000
CTR on X-ray, %	53 ± 6	54 ± 8	0.301
Atrial fibrillation, *n* (%)	3 (8)	3 (8)	1.000
AS/AR, *n* (%)	20 (53)/18 (47)	22 (58)/16 (42)	0.823
Chronic kidney disease, *n* (%)	16 (42)	14 (37)	0.815
Diabetes mellitus, *n* (%)	5 (13)	6 (16)	1.000
Preoperative echocardiographic data			
LVDd, mm	44 ± 6	44 ± 7	0.776
LVDs, mm	31 ± 8	30 ± 8	0.827
LVEF, %	55 ± 9	56 ± 8	0.593
Postoperative echocardiographic data			
Aorta area, mm^2^	1085 ± 224	745 ± 189	<0.001
Aorta diameter, mm	37.0 ± 3.9	30.6 ± 3.5	<0.001
PG max, mmHg	23.6 ± 6.5	21.7 ± 7.8	0.257
EOA, cm^2^	1.41 ± 0.26	1.37 ± 0.25	0.511
PR, mmHg	5.36 ± 1.77	6.47 ± 2.16	0.019
PRI	0.23 ± 0.06	0.30 ± 0.06	0.034
EL, mmHg	17.9 ± 5.4	14.6 ± 6.3	0.020
ELCo, cm^2^/m^2^	1.01 ± 0.27	1.06 ± 0.25	0.402

As abbreviated in Table 1, Table 2 and Table 3.

**Table 5:** Propensity-matched comparisons between AVR and Bentall groups.

Variables	AVR (*n* = 24)	Bentall (*n* = 24)	*p* Value
Patient characteristics			
Age, years	72 ± 6	71 ± 6	0.925
Gender, male, *n* (%)	16 (67)	19 (79)	0.517
CTR on X-ray, %	54 ± 7	56 ± 8	0.236
Atrial fibrillation, *n* (%)	2 (8)	2 (8)	1.000
AS/AR, *n* (%)	3 (13)/21 (87)	4 (17)/20 (83)	0.923
Chronic kidney disease, *n* (%)	8 (33)	11 (46)	0.471
Diabetes mellitus, *n* (%)	6 (25)	4 (17)	0.724
Preoperative echocardiographic data			
LVDd, mm	48 ± 9	48 ± 11	0.813
LVDs, mm	35 ± 12	36±12	0.748
LVEF, %	52 ± 10	51 ± 11	0.571
Postoperative echocardiographic data			
Aorta area, mm^2^	1088 ± 205	699 ± 115	<0.001
Aorta diameter, mm	37.1 ± 3.6	29.7 ± 2.5	<0.001
PG max, mmHg	24.5 ± 8.9	18.9 ± 7.9	0.028
EOA, cm^2^	1.40 ± 0.30	1.46 ± 0.27	0.550
PR, mmHg	5.33 ± 1.64	6.23 ± 2.95	0.022
PRI	0.23 ± 0.06	0.33 ± 0.06	< 0.001
EL, mmHg	18.7 ± 8.1	11.3 ± 4.8	0.001
ELCo, cm^2^/m^2^	1.03 ± 0.32	1.17 ± 0.32	0.168

As abbreviated in Table 1, Table 2 and Table 3.

**Table 6:** Propensity-matched comparisons between Wheat and Bentall groups.

Variables	Wheat (*n* = 21)	Bentall (*n* = 21)	*p* Value
Patient characteristics			
Age, years	74 ± 8	71 ± 6	0.180
Gender, male, *n* (%)	17 (81)	17 (81)	1.000
CTR on X-ray, %	53 ± 6	54 ± 8	0.661
Atrial fibrillation, *n* (%)	1 (5)	1 (5)	1.000
AS/AR, *n* (%)	10 (48)/11 (52)	4 (19)/17 (81)	0.100
Chronic kidney disease, *n* (%)	12 (57)	9 (43)	0.538
Diabetes mellitus, *n* (%)	3 (14)	3 (14)	1.000
Preoperative echocardiographic data			
LVDd, mm	46 ± 7	46 ± 8	1.000
LVDs, mm	33 ± 8	34 ± 8	0.884
LVEF, %	54 ± 10	53 ± 8	0.958
Postoperative echocardiographic data			
Aorta area, mm^2^	773 ± 241	700 ± 127	0.247
Aorta diameter, mm	31.1 ± 4.3	29.7 ± 2.8	0.250
PG max, mmHg	21.8 ± 7.5	18.4 ± 7.9	0.166
EOA, cm^2^	1.43 ± 0.24	1.49 ± 0.28	0.494
PR, mmHg	6.00 ± 3.04	6.48 ± 2.24	0.572
PRI	0.30 ± 0.06	0.34 ± 0.06	0.053
EL, mmHg	15.0 ± 5.9	10.8 ± 4.6	0.019
ELCo, cm^2^/m^2^	1.01 ± 0.21	1.19 ± 0.33	0.049

As abbreviated in Table 1, Table 2 and Table 3.

## Data Availability

The raw data supporting the conclusions of this article will be made available by the authors on request.
